# Oral cancer risk stratification: A cross‐sectional population‐based screening study in Northeast India

**DOI:** 10.1002/ijc.70160

**Published:** 2025-09-19

**Authors:** Kunal Oswal, Satirtha Barman, Alexandar R. Kerr, Murad Zaman, Jnyanashree Patowary, Debasis D. Barali, Nipam Barman, Ashok Das, Umakant Nadkar, Rajesh Dikshit, Jennifer E. Gallagher, Mark W. Lingen, Richard Muwonge, Philip E. Castle, Li C. Cheung, Kelly J. Yu, Anil K. Chaturvedi, Arnie Purushotham

**Affiliations:** ^1^ Karkinos Healthcare Private Limited Mumbai India; ^2^ Dr B Borooah Cancer Institute Guwahati India; ^3^ New York University College of Dentistry New York USA; ^4^ Alamelu Charitable Trusts Mumbai India; ^5^ Center for Cancer Epidemiology Mumbai India; ^6^ King's College London London UK; ^7^ University of Chicago Chicago USA; ^8^ International Agency for Research on Cancer Lyon France; ^9^ Division of Cancer Epidemiology and Genetics National Cancer Institute, National Institute of Health Bethesda USA

**Keywords:** India, oral cancer screening, risk prediction model

## Abstract

We conducted a cross‐sectional oral cancer screening study in Northeast India to develop and validate an oral precancer/cancer risk prediction model. We compared epidemiologic profiles between tobacco pouch keratosis and oral precancer/cancer. During 2018–2022, we recruited 14,749 participants who underwent an interviewer‐administered questionnaire and oral examination (visual inspection and autofluorescence). Logistic regression was used to compare risk factors between tobacco pouch keratosis and precancer/cancer and risk model development for prevalent lesions (keratosis and oral precancer/cancer, combined). Model validation was conducted internally and externally (Kerala oral cancer screening trial). Among the 14,749 participants, as per dentists' diagnosis, 1365 lesions were identified. These included 249 benign lesions (prevalence = 1.6%), 795 tobacco pouch keratosis (prevalence = 5.4%), and 321 precancers/cancers (prevalence = 2.2%). Agreement between dentists and health workers was high for visual diagnosis of prevalent lesions (keratotic/precancer/cancer; positive‐agreement = 87.5%; kappa = 0.77; 95% confidence interval [CI] = 0.75–0.78). Risk factor profiles were similar between tobacco pouch keratosis and oral precancer/cancer. The risk prediction model (based on age, sex, education, income, chewing duration, chewing type, smoking duration and intensity, alcohol duration and intensity) had good discrimination (area under the curve [AUC] = 0.83) and calibration (E/O ratio = 1.00) internally. Further, 30% of individuals at the highest model‐predicted risk accounted for 81.8% of prevalent lesions. However, in external validation, the risk model had modest discrimination (AUC = 0.67; 95% CI = 0.66–0.68) and poor calibration (E/O ratio = 0.52; 95% CI = 0.50–0.54). Our results suggest tobacco pouch keratosis as an early carcinogenic event amenable for behavioral interception. Poor transportability of our risk model reflects the need for prediction models that account for geographic differences in risk factors within regions in India.

## Graphical abstract

What's new?

Oral cancer burden is high in India. Risk stratification for selection of high‐risk individuals could enable early detection through efficient screening programs. We conducted a cross‐sectional screening study in Northeast India to develop and validate an oral precancer/cancer risk prediction model. We show that despite good internal validation, the model was poorly calibrated externally in Southern India, highlighting the need for prediction models that account for geographic differences in risk behaviors across India.

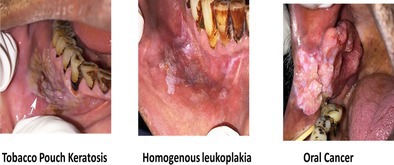

**Abbreviations**
AUC: area under the curve
BMI: body mass index
CI: confidence interval
E/O: expected/observed
OPMD: oral potentially malignant disorders
SAS: statistical analysis software

## 1 INTRODUCTION

India has one of the highest incidences of oral cancer, contributing to approximately 30% of incident cancer each year globally.^1^, ^2^ In India, oral cancer is the most common cancer among men, the third most common cancer in women,^3^ and accounts for ~23% of all cancer‐related deaths.^4^ Such high oral cancer risk arises from the high prevalence of smokeless tobacco and areca nut/betel‐quid chewing with or without tobacco.^5^ Nonetheless, there is wide heterogeneity within India in both the prevalence and the pattern of chewing and in the incidence of oral cancer, with a high oral cancer burden in Northeastern and Central India.^6^, ^7^, ^8^ This variability in chewing behaviors and oral cancer burden has implications for efficient risk‐based screening strategies in India.

Screening through visual inspection of the oral cavity significantly reduces oral cancer mortality, as demonstrated by a screening trial in India^1^, ^9^ and population‐wide screening in Taiwan.^10^ Visual and tactile examination of the oral cavity identifies cancers, precancers (leukoplakia, erythroplakia, and oral submucous fibrosis, clinically characterized as oral potentially malignant disorders [OPMDs]), as well as early mucosal changes from tobacco use (tobacco pouch keratosis).^11^, ^12^, ^13^, ^14^ Tobacco pouch keratosis (also known as smokeless tobacco keratosis) is not considered premalignant.^12^, ^14^ However, most oral premalignant lesions, and in turn cancers that progress from premalignant states, arise from the same location as tobacco pouch keratosis,^12^ highlighting such keratotic lesions as early targets for potential intervention.

The current Indian oral cancer screening guidelines recommend 5‐yearly screening in people aged 30–65 years.^15^ Such screening targets a very large fraction of the population, even when restricted to high‐risk individuals (e.g., tobacco users). For example, ~300 million ever tobacco users would currently be eligible for oral cancer screening in India.^9^ These estimates underscore the need for targeted, resource‐efficient screening strategies. The availability of strong risk factors for oral precancer and cancer makes risk‐based screening strategies feasible.^16^ Indeed, a re‐analysis of the Kerala screening trial showed that screening strategies based on predicted risk of oral cancer incidence could substantially enhance efficiency without loss of program sensitivity.^9^ For example, 50% of tobacco/alcohol users at the highest model predicted risk (23% of the full population) accounted for 73% of all oral cancers in the Kerala trial.^9^ Although oral cancer risk prediction models exist in the literature,^16^, ^17^ most do not include oral precancer and few have been externally validated.^18^ Additionally, the etiologic heterogeneity of oral cavity cancers globally mandates the development and validation of region‐specific models. Indeed, oral cavity cancers in the Americas, Europe, and Oceania are largely attributable to smoking and alcohol, while cancers in the Indian Subcontinent and other parts of Asia are attributable to chewing areca nut/betel‐quid with or without tobacco, smoking, and alcohol drinking.

We conducted a community‐based, cross‐sectional oral precancer/cancer screening study in Assam in Northeast India, a state with a high oral cancer burden owing to the high prevalence of areca nut/tobacco chewing.^19^, ^20^, ^21^ We specifically aimed to develop and validate an oral precancer/cancer risk prediction model. We also compared and contrasted epidemiologic profiles of tobacco pouch keratosis with oral precancer/cancer.

## 2 MATERIALS AND METHODS

### 2.1 Study design

During November 2018 to March 2022, we conducted a cross‐sectional study in Kamrup District of Assam, a state in Northeastern India. The study included individuals aged 30+ years who were ever chewers of smokeless tobacco and/or areca nut/betel‐quid with or without tobacco, ever smokers, or ever alcohol drinkers, and without a history of oral cancer. All participants answered an interviewer‐administered questionnaire, which captured data on participant demographics, socio‐economic status, measured anthropometrics, and tobacco/alcohol use including type, duration, intensity, and time since cessation. Participants underwent oral examination by both a dentist and a trained healthcare worker.

### 2.2 Training of examiners

Four dentists and eight healthcare workers received detailed training in the classroom from (Alexandar R. Kerr and Mark W. Lingen) which extended into the first week in the field (Alexandar R. Kerr). This included how to perform both the conventional visual examination, an autofluorescence examination, and the differential diagnosis of benign oral mucosal diseases, oral potentially malignant disorders, and oral cancer. Remote training continued with feedback on “positive” examination findings through the evaluation of digital clinical and autofluorescence images with the team (Alexandar R. Kerr).

### 2.3 Oral examination procedures

**FIGURE 1:**
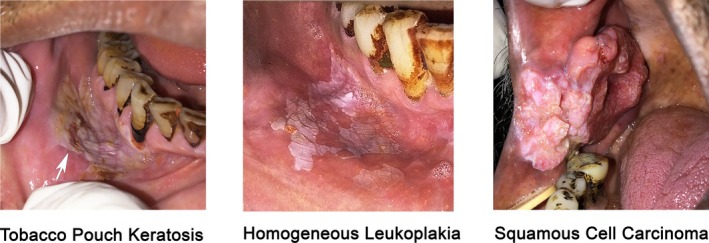
Images of representative lesions detected during the oral cancer screening study in Assam.

Each participant underwent a visual and tactile examination conducted independently by a dentist and a trained health worker. Oral mucosal abnormalities were clinically classified by each examiner as benign, tobacco pouch keratosis, or oral precancer/premalignant lesions, including lesions with a suspicion of cancer (Figure 1). Of note, we have elected to use the term oral precancer rather than OPMDs, which is primarily based on clinical features, given growing recognition in the field of cancer epidemiology for an integrative view of multistep carcinogenesis from multiple facets encompassing epidemiologic, clinical, pathologic, molecular, and genomic characteristics. Such use of precancer also aligns oral cancer with multistate carcinogenic processes at other anatomic sites. The presence of tobacco pouch keratosis and the presence and number of benign and premalignant lesions perceived were recorded. When multiple premalignant lesions were identified in an individual, clinical characteristics based on size, appearance, texture, and borders were recorded for the most severe appearing lesion. We utilized the determination by the dentists as the index lesions for analyses.

In addition to visual examination under white light, a health worker and a dentist also conducted an oral examination using autofluorescence (VelScope, LED Dental, Inc., Vancouver, Canada). Under autofluorescence at a blue light wavelength of 400 to 460 nm, normal mucosal areas retain fluorescence while abnormal areas show loss of fluorescence or hyper‐reflectance, which are common in fibrotic changes, such as oral submucosal fibrosis.^11^, ^22^ Autofluorescence characteristics of visually identified oral mucosal abnormalities deemed to be most severe were recorded as loss of fluorescence, retention of fluorescence, or hyper‐reflectance. We did not consider autofluorescence characteristics of areas that were not identified visually as abnormal.

Participants with oral premalignant lesions clinically diagnosed by dentists and 10% of participants with tobacco pouch keratosis were referred to BBCI for further investigation and biopsy. From these referred participants, at the community screening visit, we also collected 1 to 2 mL unstimulated saliva, 10 mL oral rinse samples using Scope mouthwash by three alternating rounds of swish and gargle for 5 s each, and oral cytology samples which were collected using a Rovers Orcellex cytobrush (Rovers Inc., Amsterdam, The Netherlands) and stored in SurePath medium (Becton Dickinson & Co., New Jersey, USA).

### 2.4 Statistical analyses

Participant demographic, behavioral, and medical characteristics and clinical characteristics of oral mucosal abnormalities were evaluated through descriptive statistics by percentages and mean/median, as appropriate. Comparisons between dentists and health workers utilized dentists' diagnoses as the gold standard and were conducted using percent agreement, sensitivity, specificity, and the kappa statistic. Such agreement was evaluated separately for visual examination under white light and for autofluorescence examination.

All analyses were based on dentists' diagnoses of oral precancer and tobacco pouch keratosis. Risk factor associations were estimated and compared between tobacco pouch keratosis and oral precancer/cancer as separate groups versus those with no lesions/benign lesions as the reference group, using multinomial logistic regression. These models considered age, sex, education, income, duration and intensity of smoking, chewing with or without tobacco, chewing duration, and duration and intensity of alcohol drinking. Of note, we did not consider chewing intensity because this encompasses multiple types of chewing products, making combined intensity difficult to interpret.

For the development of a risk prediction model, we used a combined group of tobacco pouch keratosis + precancer/cancer as the outcome versus those with no lesions/benign lesions as the reference group in binary logistic regression models. Further, for the continuous covariates of age, smoking duration, smoking intensity, chewing duration, alcohol duration, and alcohol intensity, we considered the best across four parameterizations, which were linear, log‐transformed, square‐root transformed, and squared. We ran multivariable models with the categorical covariates of gender, education, income, body mass index, and chewing type included in each model, and each of the four parameterizations for the six continuous covariates (*n* = 4096 models). We selected the best fitting model based on the Akaike Information Criterion.^23^

For model validation, we conducted internal 5‐fold cross‐validation. For external validation, we utilized data from the baseline screening round of the Kerala Oral Cancer Screening Trial, which was restricted to ever tobacco/alcohol users.^1^ Briefly, the Kerala trial encompassed screening through visual inspection by trained health workers, who classified oral mucosal abnormalities as referable or non‐referable lesions. Referable lesions, which we utilized for validation, included oral precancer and lesions suspicious of cancer. Metrics for model validation were discrimination, as measured by the area under the receiver operating characteristic curve (AUC), and calibration, as measured by the expected (E) to observed (O) number of cases. We also considered two model development/validation sensitivity analyses: (1) recalibration of our model to the Kerala data to account for differences in baseline risk and (2) because the Kerala trial did not consider tobacco pouch keratosis as a referable lesion, we also developed and validated a model with oral precancer/cancer as the outcome using oral precancer/cancer versus no lesions + benign lesions + tobacco pouch keratosis as the reference group.

All analyses were conducted using SAS^24^ and R Studio.^25^ *p*‐values <0.05 were considered statistically significant.

## 3 RESULTS

During November 2018 to March 2022, we recruited and screened 14,749 individuals. Participants were approximately evenly distributed by sex (Table 1). By design, almost all participants were chewers, smokers, or alcohol users (Table 1)—22.2% were ever smokers, 93.7% ever chewed (among chewers, 54.4% chewed without tobacco and 39.4% chewed with tobacco), and 25.9% were ever alcohol users. Overall, 97.4% of participants had at least one of these key oral cancer risk factors, and 13.7% of participants had all three risk factors.

**TABLE 1:** Demographic and behavioral factors among participants in a cross‐sectional oral cancer screening study in Assam, India.

Characteristic	Overall (*N* = 14,749)	No lesions/benign lesions^a^ (*n* = 13,633)	Tobacco pouch keratosis^a^, ^b^ (*n* = 795)	Precancer/cancer^a^ (*n* = 321)
	*n* (%)	*n* (%)	*n* (%)	*n* (%)
Age, years	46.7 (12.0)	46.6 (12.1)	46.9 (9.6)	47.3 (11.2)
Sex				
Men	8302 (56.3)	7330 (53.8)	731 (91.9)	241 (75.1)
Women	6447 (43.7)	6303 (46.2)	64 (8.1)	80 (24.9)
Education				
Illiterate	2029 (13.8)	1901 (13.9)	73 (9.2)	55 (17.1)
High school	6591 (44.7)	6028 (44.2)	416 (52.3)	147 (45.8)
Graduate and above	6129 (41.5)	5704 (41.8)	306 (38.5)	119 (37.1)
Income, INR per month				
<15,000	7397 (50.1)	6794 (49.8)	407 (51.2)	196 (61.1)
≥15,000	7352 (49.9)	6839 (50.2)	388 (48.8)	125 (38.9)
BMI				
<18.5	788 (5.3)	720 (5.3)	48 (6.0)	20 (6.2)
18.5–25	7693 (52.2)	7090 (52.0)	425 (53.5)	178 (55.5)
25–30	4572 (31.0)	4232 (31.0)	257 (32.3)	83 (25.9)
30+	1696 (11.5)	1591 (10.7)	65 (0.4)	40 (12.4)
Smoking status				
Never	11,477 (77.8)	10,856 (79.6)	430 (54.1)	191 (59.5)
Former	975 (6.6)	833 (6.1)	107 (13.5)	35 (10.9)
Current	2297 (15.6)	1944 (14.3)	258 (32.4)	95 (29.6)
Smoking duration, years Mean (SD)	20.1 (13.1)	20.0 (13.3)	20.2 (11.4)	22.7 (14.3)
Smoking intensity (cigarettes/day) Mean (SD)	4.7 (6.7)	4.7 (6.6)	4.4 (6.7)	5.7 (8.3)
Chewing status				
Never	923 (6.3)	913 (6.7)	3 (0.4)	7 (2.2)
Former	761 (5.2)	725 (5.3)	14 (1.8)	22 (6.9)
Current	13,065 (88.6)	11,995 (87.8)	778 (97.8)	292 (90.9)
Chewing type				
Never	923 (6.3)	913 (6.7)	3 (0.4)	7 (2.2)
Without tobacco	8022 (54.4)	7928 (58.2)	34 (4.3)	60 (18.7)
With Tobacco	5804 (39.4)	4792 (35.1)	758 (95.4)	254 (79.1)
Chewing duration, years Mean (SD)	25.7 (14.1)	25.6 (14.2)	28.3 (11.9)	26.4 (14.7)
Alcohol status				
Never	10,928 (74.1)	10,374 (76.1)	358 (45.0)	196 (61.1)
Former	635 (4.3)	536 (3.9)	80 (10.1)	19 (5.9)
Current	3186 (21.6)	2723 (20.0)	357 (44.9)	106 (33.0)
Alcohol duration, years Mean (SD)	18.7 (11.7)	18.6 (11.8)	19.4 (11.0)	18.6 (11.1)
Alcohol intensity (drinks per week) Mean (SD)	10.2 (17.8)	9.6 (17.4)	13.5 (20.2)	13.3 (18.0)

*Note*: Age was truncated at 80 years for 51 individuals; missing education (*n* = 3309 individuals) was assigned the mode; missing income (*n* = 537 individuals) was assigned the mode; missing BMI (*n* = 316 individuals) was assigned to the mode; height was truncated to be between 1 and 2 m and weight was truncated to be between 40 and 150 kg.

Abbreviation: BMI, body mass index.

^a^ Based on dentist examination.

^b^ Includes 10 individuals with smokers keratosis.

Among the 14,749 participants, as determined by study dentists clinically, 1384 lesions were identified, including 268 benign lesions (prevalence = 1.8%), 795 tobacco pouch keratosis (prevalence = 5.4%), and 321 precancers (prevalence = 2.2%) (Table 2). From 361 participants selected for biospecimen collection (all precancers excluding oral submucous fibrosis and 10% of tobacco pouch keratosis), we collected 329 (91%) (brush cytology samples, saliva, and oral rinses). Adherence with a referral for biopsy among those with referable lesions (all precancers and 10% of tobacco pouch keratosis) was 14.0% (*n* = 45). Of biopsied lesions, the prevalence of dysplasia was 8.8% (n = 4). Importantly, our screening actively identified eight cancers.

**TABLE 2:** Lesion characteristics as determined by dentists among participants in a cross‐sectional oral screening study in Assam, India.

Characteristic	*n* (%)
Lesion clinical diagnosis^a^	
Benign	268 (1.8)
Tobacco pouch keratosis^b^	795 (5.4)
Precancer	321 (2.2)
Homogeneous leukoplakia	198 (1.3)
Non‐homogeneous leukoplakia	30 (0.2)
Erythroplakia	1 (0.1)
Cancer/tumor/ulcer	18 (0.1)
Lichenoid lesion	30 (0.2)
Oral submucous fibrosis	40 (0.3)
Missing	5 (0.6)
Location (tobacco pouch keratosis + precancer/cancer)	
Labial mucosa	525 (47.0)
Tongue	9 (0.8)
Buccal mucosa	463 (41.5)
Gingiva	22 (2.0)
Palate	2 (0.2)
Floor of mouth	1 (0.09)
Retromolar trigone	2 (0.2)
Other/missing	92 (8.2)
Autofluorescence characteristics (tobacco pouch keratosis + precancer/cancer)^c^	
Retention of fluorescence	790 (70.8)
Loss of fluorescence	110 (9.9)
Hyper‐reflectance	174 (15.6)
Missing	42 (13.1)

^a^ Numbers do not add to total because of individuals having oral submucous fibrosis and other precancerous lesions.

^b^ Includes 10 individuals with smokers keratosis.

^c^ Autofluorescence characteristics were recorded for 1074 of 1116 lesions (patients with oral submucous fibrosis and without other precancerous lesions were not evaluated by autofluorescence).

As determined by study dentists, most oral precancers were homogeneous leukoplakias (61.7%), and a majority of tobacco pouch keratosis and precancers occurred at the lip and buccal mucosa, reflecting the pattern of chewing exposure (Table 2). With autofluorescence (determined by study dentists), 70.8% of visually identified mucosal abnormalities (tobacco pouch keratosis and precancers) had retention of fluorescence, 9.9% had loss of fluorescence, and 15.6% had hyper‐reflectance. Compared to tobacco pouch keratosis, precancers were significantly more likely to have loss of fluorescence (2.0% vs. 29.3%) but significantly less likely to have hyper‐reflectance (19.1% vs. 6.9%).

Compared to diagnoses by study dentists (gold standard; Table 3), health workers accurately identified the presence of tobacco pouch keratosis in 83.9% of individuals and the presence of precancer/cancer in 73.2% of individuals. Overall agreement was high between dentists and health workers for the visual identification of any lesions (tobacco pouch keratosis + precancer/cancer; sensitivity of health workers = 87.5%; specificity of health workers = 97.1%; kappa = 0.77; 95% CI = 0.75–0.78). By contrast, agreement was low between dentists and health workers for autofluorescence imaging of lesions (agreement for loss of fluorescence = 65.9%, retention of fluorescence = 89.5%, and hyper‐reflectance = 46.0%; kappa = 0.52; 95%CI = 0.46–0.58).

**TABLE 3:** Agreement between dentists and health workers for diagnosis of tobacco pouch keratosis or oral precancer/cancer through visual examination and for autofluorescence characteristics of detectable lesions.

Visual examination			
Health worker diagnosis	Dentist diagnosis (gold standard)		
	No lesions (*n* = 13,633)	Tobacco pouch keratosis (*n* = 795)	Precancer/cancer (*n* = 321)
	*n* (%)^a^	*n* (%)^a^	*n* (%)^a^
No lesions	13,238 (97.1)	90 (11.8)	46 (14.3)
Tobacco pouch keratosis	305 (2.2)	667 (83.9)	40 (12.5)
Precancer/cancer	90 (0.7)	34 (4.3)	235 (73.2)

Autofluorescence examination			
Health worker diagnosis	Dentist diagnosis (gold standard)^b^		
	Loss of fluorescence (*n* = 88)	Retention of fluorescence (*n* = 688)	Hyper‐reflectance (*n* = 163)
	*n* (%)^a^	*n* (%)^a^	*n* (%)^a^
Loss of fluorescence	58 (65.9)	45 (6.5)	5 (3.1)
Retention of fluorescence	29 (33.0)	616 (89.5)	83 (0.9)
Hyper‐reflectance	1 (1.1)	27 (3.9)	75 (46.0)

^a^ Column percentages with dental reading as the gold standard.

^b^ Autofluorescence characteristics of 939 lesions where both dentist and health worker readings were available.

Table 4 shows associations of demographic and behavioral factors with the risk of tobacco pouch keratosis and precancer. Compared to participants without lesions or benign lesions, individuals with tobacco pouch keratosis were younger, more likely to be men, have lower income, were more likely to chew areca nut with tobacco, and have a greater duration of chewing and greater duration of alcohol drinking. Similar risk factor profiles were observed in individuals with oral precancer or cancer compared to those without lesions (more likely to be men, have lower income, and more likely to chew areca nut with tobacco). Notable differences in the magnitude of risk factor associations between tobacco pouch keratosis and precancer or cancer (Table 4, *p*‐values) were observed for age (individuals with precancer were older), gender (those with precancer more likely to be women), and chewing (stronger association of chewing areca nut with tobacco for keratosis than for precancer and stronger association of chewing duration for keratosis).

**TABLE 4:** Multivariable associations of demographic and behavioral factors with risk of prevalent tobacco pouch keratosis, oral precancer/cancer, and a combined group of tobacco pouch keratosis and oral precancer/cancer.

Characteristic	Tobacco pouch keratosis versus no lesions/benign lesions	Precancer/cancer versus no lesions/benign lesions	*p*‐Value for tobacco pouch keratosis versus precancer/cancer^b^
	OR (95% CI)^a^	OR (95% CI)^a^	
Age, years	**0.96 (0.95–0.97)**	**0.99 (0.98–1.01)**	<0.012
Sex			<0.001
Men	1.00	1.00	
Women	**0.27 (0.20–0.35)**	**0.67 (0.49–0.92)**	
Education			0.37
Illiterate	1.00	1.00	
High school	1.31 (0.99–1.73)	0.87 (0.62–1.22)	
Graduate and above	1.14 (0.84–1.55)	0.93 (0.63–1.37)	
Income, INR per month			0.19
>15,000	1.00	1.00	
≥15,000	**0.84 (0.71–0.99)**	**0.62 (0.48–0.81)**	
Body mass index (BMI)			0.13
<18.5	1.00	1.00	
18.5–25	1.03 (0.74–1.44)	1.11 (0.69–1.79)	
25–30	0.98 (0.70–1.39)	0.92 (0.55–1.53)	
30+	0.90 (0.59–1.36)	1.44 (.82–2.54)	
Smoking duration, years	1.00 (0.99–1.01)	**1.02 (1.00–1.03)**	0.14
Smoking intensity (cigarettes per day)	1.00 (0.98–1.02)	1.01 (0.99–1.04)	0.62
Chewing type			<0.001
Never	1.00	1.00	
Without tobacco	1.01 (0.30–3.36)	1.26 (0.54–2.96)	
With tobacco	**23.4 (7.3–74.5)**	**7.2 (3.2–16.2)**	
Chewing duration, years	**1.03 (1.02–1.04)**	1.00 (0.99–1.01)	0.007
Alcohol duration, years	**1.01 (1.00–1.02)**	0.99 (0.98–1.01)	0.052
Alcohol intensity (drinks per week)	1.01 (1.00–1.01)	1.00 (0.99–1.01)	0.89

*Note*: Values in bold are statistically significant at *p* < 0.05.

Abbreviations: CI, confidence interval; OR, odds ratio.

^a^ Results from multinomial logistic regression models with tobacco pouch keratosis and precancer/cancer as separate outcomes. Models simultaneously adjusted for all covariates in the table.

^b^ *p*‐Values estimated in binary logistic regression models restricted to individuals with tobacco pouch keratosis (*n* = 795) or oral precancer/cancer (*n* = 321).

Table 5 shows the best‐fitting risk model for the prediction of prevalent tobacco pouch keratosis and precancer/cancer (combined as one outcome). Prevalence of lesions was statistically significantly associated with younger age, male gender, lower monthly income, greater duration of smoking and chewing, and chewing areca nut with tobacco. The prediction model had good discrimination (AUC = 0.83) and calibration (E/O = 1.00) in internal 5‐fold cross‐validation. Using this prediction model within the same study population (Table S1, Supporting Information), 30% of individuals at highest model predicted risk included 81.8% of prevalent tobacco pouch keratosis and precancer/cancer.

**TABLE 5:** Risk prediction model for prediction of prevalent tobacco pouch keratosis and precancer/cancer.

Characteristics	Coding	Tobacco pouch keratosis + precancer/cancer	Tobacco pouch keratosis + precancer/cancer	*p*‐Value
		Beta coefficient	OR (95% CI)^a^	
Age, years	Squared	−0.00036	**1.00 (1.00–1.00)**	<0.001
Gender	Categorical			<0.001
Men		0.00	1.00	
Women		−0.9160	**0.40 (0.32–1.49)**	
Education	Categorical			0.49
Illiterate		0.00	1.00	
High school		0.0912	1.10 (0.88–1.37)	
Graduate and above		0.0145		
Income, INR per month	Categorical			<0.001
<15,000		0.00	1.00	
≥15,000		−0.2697	**0.76 (0.66–0.80)**	
Body mass index	Categorical			0.66
<18.5		0.00	1.00	
18.5–25		0.0408	1.04 (0.79–1.38)	
25–30		−0.0532	0.95 (0.70–1.28)	
30+		0.0369	1.04 (0.73–1.47)	
Smoking duration	Linear	0.00952	**1.01 (1.00–1.02)**	0.001
Smoking intensity (cigarettes per day)	Squared	0.000166	1.00 (1.00–1.00)	0.39
Type of chewing	Categorical			<0.001
Never		0.00	1.00	
Without tobacco		−0.6851	0.50 (0.24–1.05)	
With tobacco		1.8236	**6.19 (3.07–12.52)**	
Chewing duration	Square‐root	0.2495	**1.28 (1.20–1.37)**	<0.001
Alcohol duration	Natural log	0.0312	1.03 (0.97–1.10)	0.35
Alcohol intensity (drinks per week)	Square‐root	0.0438	1.05 (1.00–1.09)	0.052

*Note*: Values in bold are statistically significant at *p* < 0.05.

Abbreviations: CI, confidence interval; OR, odds ratio.

^a^ Results from binary logistic regression models with tobacco pouch keratosis and precancer/cancer as a combined outcome and no lesions/benign lesions as the reference group. Models simultaneously adjusted for all covariates in the table.

However, in external validation with the Kerala trial baseline data, the risk model had moderate discrimination (AUC = 0.67; 95% CI = 0.66–0.68) and was poorly calibrated (E/O = 0.56; 95% CI = 0.54–0.58). Recalibration of the risk model to the Kerala data did not improve discrimination. The use of a risk model to predict only precancer/cancer had similar results (moderate discrimination and poor calibration, not shown).

## 4 DISCUSSION

We present key observations from a cross‐sectional oral cancer screening study in Assam, India, a region with notably high oral cancer incidence due to the high prevalence of smokeless tobacco and areca nut chewing (with or without tobacco).^26^ First, there was high agreement between dentists and trained health workers for the identification of oral lesions (tobacco pouch keratosis and precancer/cancer) through visual and tactile examination, underscoring the scalability of oral cancer screening in resource‐limited settings. Second, risk factor profiles were very similar for tobacco pouch keratosis (not generally considered a premalignant state) and oral precancer, suggesting that tobacco pouch keratosis could represent an early form of oral precancer amenable to interception. Third, a risk prediction model developed in Northeast India with good performance internally had poor transportability to South India, reflecting the need for risk prediction models that accommodate the geographic variability in risk behaviors within India.

We found high agreement (~87.5%) between trained health workers and dentists for the identification of oral lesions (tobacco pouch keratosis or precancer/cancer), similar to prior studies.^27^ Yet, agreement, while high for tobacco pouch keratosis (~83.9%), was somewhat lower (~73.2%) for the identification of oral precancers/cancers. These results indicate that trained health workers could be used to identify individuals with referable oral mucosal lesions (tobacco pouch keratosis and precancer, cancer), with additional triage of lesions (e.g., for biopsy) conducted by dentists/experts.^28^, ^29^, ^30^ By contrast, we found that agreement between health workers and dentists was low for the determination of autofluorescence characteristics, arguing against its utility for primary screening by health workers. Further, a combination of one or more factors inherent among chewers of areca nut/betel‐quid, such as extrinsic staining of the oral mucosa (betel‐chewer's mucosa), reactive melanosis, and hyper‐reflectance, reduces the utility of autofluorescence in Asian populations.^31^

The potential feasibility and scalability of oral cancer screening by health workers notwithstanding, the large population (1.4 billion) and high prevalence of risk behaviors in India (>300 million ever users of cigarettes or tobacco/areca nut chewing) indicate the need for risk stratification for the selection of individuals for screening.^9^ Such risk stratification could be accomplished through the use of individualized risk prediction models.^9^, ^17^, ^18^ Our results highlight the poor transportability of risk prediction models across geographic regions in India. We hypothesize that such poor transportability, in part, arises from wide variability in patterns of tobacco/areca nut chewing across India.^8^ Indeed, according to the Global Adult Tobacco Survey (2016–2017),^8^ diverse products and patterns of chewing are prevalent across India (Tamul, khaini, zarda, pan masala, dentobacc, misheri, and gudaku).^8^ In Assam, Khaini (23.1%) and betel quid with tobacco (19%) are the two most common tobacco products, whereas in Kerala, cigarette (6.7%) and betel quid with tobacco (4.4%) are the two most common tobacco products.^8^ Thus, our results underscore the need for oral cancer risk prediction models that accommodate variability in risk behaviors across India or region‐specific data that allow recalibration of models developed in other regions in India.

Few studies have compared risk factor profiles of tobacco pouch keratosis, which is generally not considered a premalignant lesion, and oral precancers (leukoplakia, erythroplakia, oral submucosal fibrosis).^12^, ^14^ Our results highlight strong parallels in risk factor profiles between tobacco pouch keratosis and oral precancer, underscoring such keratotic lesions as potentially earlier forms of premalignant lesions amenable to interception through counseling for cessation of risk behaviors. Indeed, there is growing recognition in the field that tobacco pouch keratosis should be considered a referable lesion akin to oral leukoplakia/erythroplakia.^14^, ^32^, ^33^, ^34^ We note that our study only compared epidemiologic profiles between tobacco pouch keratosis and oral precancer, and additional studies are needed to compare the pathologic and genomic profiles of tobacco pouch keratosis and oral premalignant lesions.

Our results need to be interpreted within the context of the study limitations. Our diagnosis of oral precancer was primarily based on clinical/visual impression rather than biopsy‐driven histopathology because there was very low compliance with biopsy recommendations (14.0%). This limitation, unfortunately, is pervasive in oral cancer screening studies.^1^, ^28^, ^35^ Such low compliance could have also arisen, in part, due to the COVID‐19 pandemic, as our field effort spanned 2018 to 2022. One of the fundamental problems in cancer screening in India is convincing people to continue down the diagnostic and treatment pathway even when information is communicated, and the infrastructure, human resources, and transport are in place to make it easier for patients to reach the diagnostic and treatment centres, as was facilitated in this study. The reasons for this reluctance are multifactorial and related to fear, stigma, geographical distance, loss of income, financial toxicity, gender discrimination, and religious reasons, among others. The results of our study further highlight this problem and emphasize the need to address the psycho‐social barriers to accessing diagnostics and treatment when required. Further qualitative studies are needed to characterize barriers and enablers for adherence to referral recommendations among screen‐positive individuals. It is also important to ensure that the infrastructure and trained human resources are in place to diagnose and treat patients in whom oral precancer/cancer is suspected. We found that the discrimination of our risk prediction model was moderate. The almost exclusive inclusion of ever smokers/chewers in our study could have made our study population more homogeneous in risk, curtailing discrimination from duration and intensity of smoking/chewing. The strengths of our study include the large sample size, novel comparisons of risk profiles between tobacco pouch keratosis and oral precancer, and the use of internal and external validation of our risk prediction model.

The Global Strategy and Action Plan on Oral Health 2023–2030 defines World Health Organization's^36^ global oral health agenda. Together, the policy documents describe the path to tackle the challenges faced globally and advocate integrating oral health into noncommunicable diseases and universal health coverage benefit packages.

In summary, our study points to the feasibility and scalability of oral cancer screening conducted by trained health workers in India. Our results also indicate the potentially poor transportability of risk prediction models to different regions within India, which highlights the need for models that accommodate the variability in oral cancer risk behaviors across India.

## AUTHOR CONTRIBUTIONS

**Kunal Oswal:** Conceptualization; methodology; funding acquisition; writing – original draft; writing – review and editing; formal analysis; project administration; data curation; supervision; resources; investigation. **Satirtha Barman:** Conceptualization; investigation; writing – original draft; methodology; writing – review and editing; formal analysis; project administration; data curation; supervision; resources. **Alexandar R. Kerr:** Conceptualization; writing – original draft; writing – review and editing; methodology; supervision; resources; investigation. **Murad Zaman:** Investigation; writing – review and editing; methodology; project administration; resources. **Jnyanashree Patowary:** Investigation; methodology; writing – review and editing; project administration; resources. **Debasis D. Barali:** Investigation; methodology; writing – review and editing; project administration; resources. **Nipam Barman:** Investigation; methodology; writing – review and editing; project administration; resources. **Ashok Das:** Investigation; methodology; writing – review and editing; project administration; supervision; resources. **Umakant Nadkar:** Investigation; methodology; writing – review and editing; project administration; supervision; resources. **Rajesh Dikshit:** Conceptualization; investigation; writing – review and editing; methodology; formal analysis; project administration; data curation; supervision; resources. **Jennifer E. Gallagher:** Conceptualization; funding acquisition; writing – review and editing; methodology; resources. **Mark W. Lingen:** Methodology; writing – review and editing; supervision; conceptualization; resources. **Richard Muwonge:** Methodology; validation; software; formal analysis; project administration; data curation; writing – review and editing. **Philip E. Castle:** Conceptualization; writing – review and editing; methodology; funding acquisition; project administration; resources. **Li C. Cheung:** Conceptualization; investigation; writing – review and editing; methodology; software; formal analysis; data curation; validation. **Kelly J. Yu:** Conceptualization; investigation; funding acquisition; writing – original draft; writing – review and editing; methodology; formal analysis; project administration; supervision; resources; validation. **Anil K. Chaturvedi:** Conceptualization; investigation; funding acquisition; writing – original draft; methodology; validation; writing – review and editing; formal analysis; project administration; supervision; data curation; resources. **Arnie Purushotham:** Conceptualization; investigation; funding acquisition; writing – original draft; methodology; writing – review and editing; formal analysis; project administration; supervision; resources; validation; data curation.

## CONFLICT OF INTEREST STATEMENT

The authors declare no conflict of interest. Where authors are identified as personnel of the International Agency for Research on Cancer/World Health Organization, the authors alone are responsible for the views expressed in this article and they do not necessarily represent the decisions, policy, or views of the International Agency for Research on Cancer/World Health Organization.

## ETHICS STATEMENT

The study was reviewed and approved by the ethics committee of the Bhubaneswar Borooah Cancer Institute, a regional multispecialty cancer center under the aegis of the Tata Memorial Center, and registered under the Clinical Trial Registry of India–BBCI/TMC/Misc‐119/3982/2018.

## Supporting information


**Table S1.** Prevalence of tobacco pouch keratosis and oral precancer/cancer across risk thresholds internally in the cross‐sectional screening study population.^a^

## Data Availability

The data that support the findings of this study are available from the corresponding author upon reasonable request.
