# Supplementary material for: Oral cancer risk stratification: A cross‐sectional population‐based screening study in Northeast India
Source: Int J Cancer. 2025 Sep 19;158(6):1517–27. doi: 10.1002/ijc.70160 (PMC12811214; doi:10.1002/ijc.70160)
Supplement: Supplementary file 1 — Table S1. Prevalence of tobacco pouch keratosis and oral precancer/cancer across risk thresholds internally in the cross‐sectional screening study population.a [file IJC-158-1517-s001.pdf]

Supplementary File for

Oral cancer risk stratification: a cross-sectional population-based screening study in Northeast India

Kunal Oswal, Satirtha Barman Alexandar R. Kerr, Murad Zaman, Jnyanashree Patowary, Debasis D Barali, Nipam Barman, Ashok Das, Umakant Nadkar, Rajesh Dikshit, Jennifer E Gallagher, Mark W. Lingen, Richard Muwonge, Philip E. Castle, Li C. Cheung, Kelly J. Yu, Anil K. Chaturvedi, Arnie Purushotham

Supplementary Table 1. Prevalence of tobacco pouch keratosis and oral precancer/cancer across risk thresholds internally in the cross-sectional screening study population [a]

Risk quintile	Prevalence of tobacco pouch keratosis and oral precancer/ cancer (n=1116)
1st decile (lowest, 0-10% of model-predicted risk)	1.0%
2nd decile (10-20%)	0.8%
3rd decile (20-30%)	1.1%
4th decile (30-40%)	1.3%
5th decile (40-50%)	1.3%
6th decile (50-60%)	3.1%
7th decile (60-70%)	9.6%
8th decile (70-80%)	19.1%
9th decile (80-90%)	25.2%
10th decile (highest, 90-100% of model-predicted risk)	37.5%

[a] See Table 5 for variables included in the risk prediction model. Model developed and applied internally in the oral cancer screening study (N=14749 participants)
